# Editorial

**Published:** 1860-04

**Authors:** 


### Editorial.

### "PRACTICAL HINTS."

The first article in the March No. of the Cosmos, entitled "Practical Hints"—by J. D. White, contains some rather peculiar ideas upon a very important operation, namely the method of plugging the roots of teeth. Dr. W. remarks: "In recent cases, there is frequently a fragment of the blood-vessel and nerve left in the lower third or fourth of the fang, out of the reach of our instruments, and to thrust the gold against this, would be to excite inflammation of the external periosteum." This is very true, but it is equally as true, that inflammation of the periosteum will occur, if a space is left in the canal, between the filling and the remaining portion of the pulp. In the great majority of cases, that space will be filled by a discharge from the nerve, and this will produce equally as great difficulty, as though the gold were in contact with the living fragment; if any portion of the canal is left unfilled, it might as well all be so. "We never enlarge the natural canal of the fang, to follow a pulp, to the apex of the root." Then we think you fail in some cases, to do your duty. We think the true method in all such cases, is to remove all remaining portions of the pulp. The fact that we can not with the usual instrument reach a fragment of pulp through the canal in the natural condition, is no argument why it should not be removed; it would be just as admissable to leave a portion that could be approached—almost every canal may be so enlarged, that all fragments of the pulp can be removed to the termination of the canal. Every such fragment of a pulp, has lost its function, and can not with impunity remain. Nature endeavors to throw off every such part. We do not think that nature will take care of such a part better than we can.

How will nature take care of it? What will she do with it? Well in nine cases in ten it will slough off, or constantly discharge pus, and the vacuum be filled up with vitiated matter that will produce irritation, inflammation and abscess. Now if the canal was entirely filled, nothing of this kind could take place, from this cause at least. The Doctor proceeds to describe his method of filling fangs, which probably in his hands is very good, but one thing is very clearly set forth, that is, that he makes provision for ensuing trouble. This is probably a very wise arrangement; considering the treatment he gives the fangs. We should expect if we treated fangs in the manner he describes, to remove the fillings from almost all the roots, and we think his precaution to put his plugs in so he can pull them out, a very wise one. But it would certainly be far better to treat the cases in such a manner that no such necessity would arise.

"There are other methods of introducing gold, but as we never resort to them, it is useless to enumerate them." What a sentence! If *some* men had used this language, we should say it had a sprinkling of egotism. Is it possible that any method, "we never resort to," is not worth enumerating? Do "we" practice the only method that is worth any thing? Are those plugs that can be stuck in, and pulled out at pleasure, the only ones worth referring to? We would suggest, that you might "enumerate" some other methods, and then "resort" to them, (especially in the treatment of fangs) without suffering materially thereby. Again: "We never attempt to plug the roots of molar teeth, unless we can get into them with facility, except it is in old cases where the crowns are broken away." How is this? Never attempts to plug the roots of molar teeth he can't get into with facility, except in old cases where the crowns are broken away? Now this is either funny or ridiculous, we are not certain which. Why sir, you can get into the fangs of any tooth and with facility too, if you pursue the proper course. We do not exactly see the propriety of filling the roots of old cases, where the crowns are broken away, whether they can be approached with or without facility. "We content ourselves with filling the bulbous portion only of the pulp cavities; it is in front teeth, canines and bicuspids that we are careful." You only fail in not adding the molars to this list, you had better be "careful" with all the teeth, and especially with molars having good crowns, as well as in the old cases where the crowns are broken away.

We refer to this article of Dr. White's because we consider it radically wrong in its teachings, and one calculated to lead young members of the profession into error. It will never injure any one who has adopted the right principle, and carried it into practice, but it is those who are seeking the truth, that are liable to be led astray. T.

### OS-ARTIFICIAL.

This preparation for filling teeth has been before the profession for some time, though not very extensively tried. We have been for sometime using this preparation as prepared by Dr. C. H. Roberts, and it accomplishes more than we had anticipated; we deem it proper to refer to some of its more prominent features. And first, it seems to be well adapted for temporary fillings, whenever they are required. It will serve a good purpose for filling the deciduous teeth. It is the best preparation we have seen for temporary filling where there is sensitive dentine; it protects the cavity effectually, and the caustic property reduces the sensitivenesss. From one to five days, according to experiment, will be quite sufficient with a filling of this kind to render sensitive dentine sufficiently normal to receive a gold filling. It is sufficiently a non-conductor for this purpose.

Again, it is valuable for filling teeth that are very much decayed, and for building up crowns that are partially broken away. It is valuable in this respect only, for molars and bicuspids. The crowns of the teeth may be built up to their original size and form. Of course it will wear down with more or less rapidity, according to the force with which the teeth are used, but it seems to be sufficiently firm to wear for a considerable time probably from two to five years. It attaches itself to the walls of the cavity, so that it will remain till it is either cut or worn away—will not come out in pieces. It is not necessary to make retaining pits for it, nor even to have the cavity of a general retaining form, for it adheres sufficiently firm to retain its position without. It may be used in the preparation of a root that is much decayed, for the reception of a pivot tooth; it may also be used for attaching the gums of teeth, which may be accidentally broken from an artificial denture. Notwithstanding we think it a good thing, there are some points about it which need watching. The fluid is very caustic, and if it comes in contact with the soft parts, before it is thoroughly combined with the powder, it produces a very unpleasant taste and a painful sensation, and cauterizes the surface. We think it would not be proper to fill a tooth, the pulp of which is exposed, with it, if we wish to preserve its vitality, without at least placing a shield over it, though in this particular we have not much experience.

It can not in any case be recommended for permanent fillings. It should not be used to fill ordinary cavities that can be filled with gold. The method of applying it is very simple, and is fully described in the directions accompanying it. It is worked in with a fine sharp pointed spatulæ, having about the consistence of putty. The cavity should be thoroughly dried, and the filling after being introduced should be kept free from moisture from five to twenty minutes ; a jet of warm air facilitates the hardening process. Time and experiment only, can test its value. T.

### SCIENTIFIC ARTISAN.

This publication, to which we have before referred, is one which we peruse with a great deal of pleasure and profit.

The subjects discussed, explained and clearly set forth, are such as are, or should be highly interesting to all who feel any interest in the various departments of science, art and agriculture. It enters freely and fully into the subjects involved in these three departments. No one who desires to keep posted in these various departments, (and who should not?) can afford to be without this or some similar publication. Every one, whether a farmer, a professional man or day laborer, may by the weekly perusal of such a paper, in a very little while become an accomplished scientifician. He is constantly attaining knowledge which he can make tributary to his every day operations. The subjects are treated so familiarly that the ideas can at once be comprehended and applied to practical purposes.

A common fault with most of our scientific works is, that the ideas are so loaded down with verbiage, that it costs as much as they are worth to dig them out, but a reform is taking place, and we are pleased to see the Scientific Artisan leading the way.

Justice requires us to say, however, that the Scientific American is going hand in hand with the Artisan. No physician, or lawyer, or minister, or dentist, or mechanic, or farmer, or day laborer, can afford to be without such a paper as the Artizan, if he desires to know what is going on in the world of art and science—and all should know. T.

### CINCINNATI DENTAL SOCIETY.

The regular monthly meeting of this society was held on Tuesday evening the 13th of March, at the office of Drs. Wardle & Doughty. Quite a number of the members of the profession in the city, were present. This being the regular time for the election of officers, they were chosen for the next half year. There was no essay read, the essayist not being present.

The topic for discussion, "Hemmorrhage after extracting teeth, its causes, nature and treatment," was taken up, and the views of the members pretty fully given—the discussions we will probably give in the next number of the Register. Some new and interesting ideas were elicited. Two or three particulars in regard to modes of practice were referred to.

Drs. H. R. Smith and T. F. Davenport were appointed essayists for the next meeting.

Filling teeth is the subject designated for discussion. We expect to get something new on this important subject. We hope the committee on the tobacco question will be ready to report.

The society made an appropriation of twenty-five dollars to aid in the purchase of a microscope in connection with the Mississippi Valley Association.

Adjourned to meet at the office of Drs. Taft & Foote, the second Tuesday in April, at 7½ o'clock, P. M.

All members of the profession are cordially invited to attend.

---

CLEVELAND, Ohio, March 6th, 1860.

In answer to a call, the nucleus of Northern Ohio Dental Association, formed last May, met at 2 o'clock, P. M., March 6, for organization, adoption of a constitution and rules of regulation.

After the object of the meeting was stated by the chairman, Dr. B. Strickland, on motion and by a unanimous vote, several others who had been invited were elected to take part in the organization of a society to facilitate professional intercourse.

After which a constitution and a code of by-laws, were presented, read, and after slight modifications were adopted.

On motion the association then proceeded to the election of officers for the ensuing year, which resulted as follows:

Dr. B. Strickland, President, Cleveland, O.

Dr. H. P. Hunting, Vice-President, Painesville, O.

Dr. C. R. Butler, Recording Secretary, Cleveland, O.

Dr. B. F. Robinson, Corresponding Secretary, Cleveland, O.

Dr. F. S. Slossen, Treasurer, Cleveland, O.

Drs. W. H. Atkinson, Cleveland, O.; C. Palmer, Warren, O.; B. H. Spelman, Ravenna, O., Examining Committee.

On motion, the Secretary was instructed to send a synopsis of the proceedings of the meeting for publication.

Adjourned to meet in Cleveland, the first Tuesday in May, 1860, 7½ o'clock, P. M. C. R. Butler, D. D. S., Secretary.

### LITTLE MIAMI RAILROAD.

This pioneer road continues to be a universal favorite. We give below the time of the arrival and departure of its trains, for the convenience of those of our readers who may desire to travel on it.

Fewer accidents have occurred upon this road than upon any other with which we are acquainted. This is the result of its superior management.

Regular Passenger Trains Leave—10 A. M., Day Express; 4:40 P. M., Accommodation; and 11:30 P. M., Through.

Regular Passenger Trains Arrive—7:30 A. M., 1:25 P. M., and 10:45 P. M. Makes regular connections with the Hillsboro' Road Loveland, and the Wilmington and Zanesville Road at Morrow.

### BACK NUMBERS.

We wish to procure a few copies of the first number of Volume XII., of Dental Register, (September of 1858.) We will pay $1 00 each, for a few copies. Any person having this number and willing to spare it, will please mail it to the "Dental Register of the West," Cincinnati, O., with their name and place written upon it, that we may know from whom it came. T.

